# Site-Specific Functionalization of Recombinant Spider Silk Using Enzymatic Sortase Coupling

**DOI:** 10.1021/acsomega.4c09900

**Published:** 2025-02-06

**Authors:** Rajeev Pasupuleti, Ronnie Jansson, Ida Isacsson, Felicia Hogan, Mona Widhe, My Hedhammar

**Affiliations:** KTH Royal Institute of Technology, School of Engineering Sciences in Chemistry, Biotechnology and Health, Department of Protein Science, AlbaNova University Center, Roslagstullsbacken 21, SE-106 91 Stockholm, Sweden

## Abstract

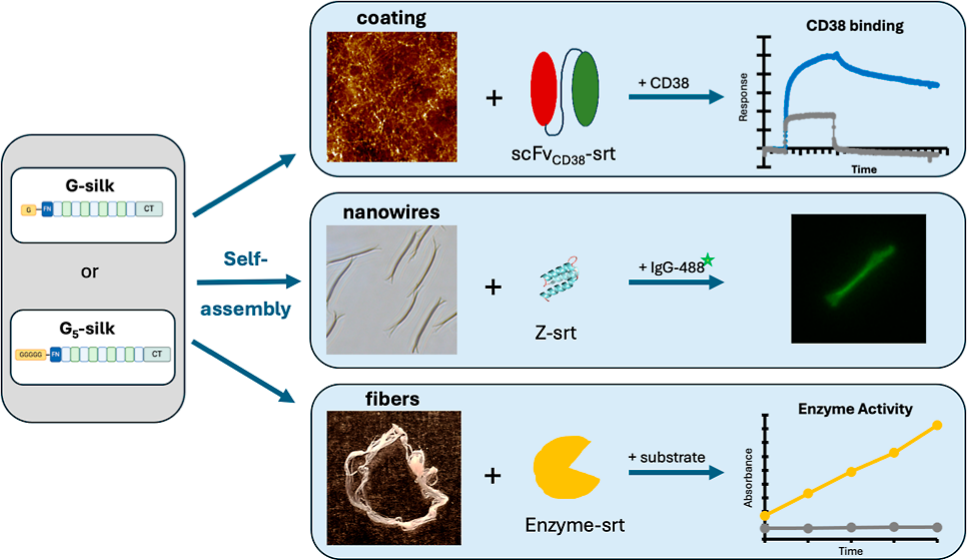

Functionalization of biomaterials with extra protein domains will expand their functional roles in biomedical research. The recombinant spider silk protein FN-4RepCT has been shown able to adapt various formats like coatings, nanowires, and macroscopic fibers. Functionalizing these various formats of FN-4RepCT in a site-specific manner will provide the next generation of biomaterials. The current study reports an enzymatic (sortase A) coupling method to site-specifically functionalize various formats of FN-4RepCT with target proteins. The approach is demonstrated with three different functional proteins: the IgG-binding Z-domain, a single-chain variable fragment with specificity for CD38 (scFv_CD38_), and the antibacterial endolysin Sal-1. The target proteins were produced with an LPETGG sortase recognition tag at the C-terminus to enable coupling. Moreover, a comparative analysis of sortase coupling efficiency of the target proteins was performed using two different silk protein variants, FN-4RepCT with one N-terminal glycine (G-silk) and five N-terminal glycines (G_5_-silk). The functionalized silks were assessed by using protein gel electrophoresis, fluorescence microscopy, surface plasmon resonance, and a biochemical assay. Results showed that G_5_-silk is more efficient for sortase coupling of the target proteins in solution as well as to silk coatings, when compared to G-silk. In all cases, the target proteins, the Z-domain, the scFv_CD38_ fragment, and Sal-1, retained their specific activity after sortase coupling. To conclude, the sortase coupling strategy is a mild and efficient approach to functionalize various silk formats with small (Z-domain) or larger (scFv_CD38_, Sal-1) functional molecules.

## 1 Introduction

Recombinant spider silk is emerging as a potential material for use in, for example, tissue engineering^1,2^ and drug delivery^3,4^ due to the biocompatibility, slow biodegradation, low immunogenicity, and excellent mechanical properties.^1,5,6^ One of the extensively studied recombinant spider silk proteins is the 4RepCT, which is a miniature form of the major ampullate spidroin 1 (MaSp 1) from *Euprosthenops australis*.^5^ The addition of an extra motif or domain to the silk proteins enhances their functional roles in downstream applications of biomedical research. Hence, 4RepCT has been established as a bifunctional molecule when genetically fused to cell-adhesion peptides (RGD, IKVAV, and YIGSR), IgG-binding domains (Z and C2), and xylanase, to form cell binding silk,^7^ affinity silk,^8^ and enzyme silk,^9^ respectively. In addition, the 4RepCT silk protein, and functionalized variants thereof, can be transformed into various formats like coating,^10^ foam, mesh, film,^11^ membranes,^12^ and macroscopic silk fibers,^5,13^ expanding its scope to a broad range of biomedical applications. However, functionalization of proteins by genetic fusion in bacterial production systems is not always possible, for example, if the fusion partner is a small organic molecule, a nucleic acid, or a protein, which requires post-translational modifications.^14−16^ Moreover, while functionalizing 4RepCT on the gene level, the fusion partner might interfere with the self-assembly during formation of the above-mentioned silk formats. Therefore, the current aim was to develop a sortase A-based coupling method as an additional strategy to functionalize 4RepCT instead at the protein level. This approach might also enable us to add functional protein domains to the already formed 4RepCT silk formats. In the present study, the FN-4RepCT silk variant, with a cell binding motif from fibronectin (FN), is used and, thus, after sortase ligation to the new functional domain, it will attribute the silk with double functional properties. The FN-4RepCT contains one N-terminal glycine, G-FN-4RepCT (from now on denoted G-silk), and is herein compared with a variant with five N-terminal glycines, G_5_-FN-4RepCT (from now on denoted G_5_-silk).

Anchoring surface proteins to peptidoglycans in bacteria is the basis for sortase A coupling found in nature. Sortase A cleaves the bond between threonine and glycine of the C-terminal sortase recognition tag, LPXTG (X is any canonical amino acid), and catalyzes a covalent linkage by an amide bond with an N-terminal glycine.^17,18^ Chemo-enzymatic ligations using sortase have been reported for the production of bispecific antibodies^19^ and immobilization of enzymes^20^ in a site-specific manner at the protein level. In our recently published article, we reported sortase-mediated ligation of antimicrobial enzymes to silk coatings of 4RepCT.^10^ To investigate the concept further, the enzymatic ligation with sortase is repurposed to covalently ligate three different formats of G-silk and G_5_-silk with the immunoglobulin-G (IgG)-binding Z-domain, a single-chain variable fragment (scFv), and the endolysin Sal-1.

The Z-domain is a mutated version of the B-domain of Staphylococcal Protein A which has a three-helix bundle structure and competently binds to the Fc region of IgG.^21^ The herein used scFv_CD38_ is specific to the cell surface expressed receptor CD38, which is overexpressed in, e.g., multiple myeloma and acute leukemia.^22^ Bispecific T-cell engagers constituting anti-CD38 Fab/anti-CD3 scFv have been shown to be able to promote T-cell-specific killing of myeloma cells.^23^ Therefore, we considered the Z-domain and scFv_CD38_ to be suitable examples of modules to be ligated to silk, enticing their role in diagnostics and targeted drug delivery. By the current report, we present the possibility of protein level covalent coupling of the Z-domain or scFv_CD38_, both equipped with a C-terminal sortase recognition tag (Srt) LPETGG, to the N-terminus of the G-silk and G_5_-silk proteins using an optimized recombinant variant of the Sortase A enzyme. First, sortase coupling was performed in solution and confirmed by sodium dodecyl sulfate polyacrylamide gel electrophoresis (SDS-PAGE). As there is a growing interest in attaching proteins to coatings and solid materials,^17,18^ sortase coupling of the Z-domain to coatings and nanowires of G_5_-silk was also investigated. The functionality of the coupled Z-domain was assessed by IgG-binding studies using fluorescence microscopy and Surface Plasmon Resonance (SPR). Finally, an endolysin, Sal-1, was ligated to the fiber format of G_5_-silk to prove the strategy for coupling of larger molecules with enzymatic activity onto macroscale silk materials.

## 2 Experimental Section

### 2.1 Design of Recombinant Proteins

To generate a plasmid containing the DNA sequence corresponding to G_5_-silk, the synthetic sequence Trx-His6-TEVp-G_4_ was ordered (Thermo Fisher Scientific Inc., Rockford, IL). The solubility tag Trx is short for Thioredoxin, and TEVp represents the recognition sequence for Tobacco etch virus (TEV) protease (ENLYFQG, TEV protease cleaves between Q and G) and His6 for the hexahistidine tag. The synthetic sequence Trx-His6-TEVp-G_4_ was cloned into the target plasmid His6-X-G-FN-4RepCT using the restriction enzymes NdeI and EcoRI generating the plasmid encoding His6-Trx-His6-TEVp-G_5_-FN-4RepCT (for sequence, see Supporting Information Table S1).

The sequence for scFv_CD38_ was designed by fusing the amino acid sequences for variable heavy, *V*_H_, and light, *V*_L_ (sequences kindly provided by Dr. Carin Dahlberg), with a 3xG_4_S linker in-between. Reverse translation was then performed on the generated scFv_CD38_ amino acid sequence, followed by addition of the restriction cleavage sites NcoI and NotI to the N- and C-terminus of scFv_CD38_, respectively. The entire DNA sequence was then ordered as a synthetic gene (Thermo Fisher Scientific). The synthetic scFv_CD38_ gene fragment was cloned into the target plasmid pET-26b, which has a sortase recognition tag, LPETGG, followed by a His6 tag, generating a sequence corresponding to scFv_CD38_-Srt-His6. This plasmid was then used for further subcloning of the scFv_CD38_-Srt-His6 sequence into the target plasmid, p1 hpc, for subsequent expression of the scFv_CD38_-Srt-His6 protein (for sequence, see Supporting Information Table S1) in Chinese Hamster Ovary (CHO) cells.

The two individual plasmids coding for the protein, Z-Srt-His6 (plasmid denoted as pAY430-ZWT-SR-H6), and for the sortase A enzyme, variant P94S/D160N/K196T (plasmid denoted as pGBMCS-P96D/D160N/K196T-SortA),^24,25^ were kindly provided by Dr. Kristina Westerlund (for sequences, see Supporting Information Table S1).

### 2.2 Expression and Purification of Proteins

Both of the silk proteins used in this study, G_5_-FN-4RepCT (G_5_-silk) and the G-FN-4RepCT (G-silk), were kindly produced by Spiber Technologies AB, Stockholm, SE. Soluble silk proteins were provided as frozen aliquots that were stored at −80 °C upon arrival until use. The silk proteins were first thawed and then centrifuged for 2 min (13,000 rcf, +4 °C) before use. The sortase A variant P94S/D160N/K196T (from now on denoted sortase or Srt A) and the Z-Srt-His6 protein (from now on denoted Z-domain) were produced as previously described^26^ using expression in *Escherichia coli*, followed by purification by standard immobilized metal ion affinity chromatography (IMAC). The scFv_CD38_ protein was produced in CHO cells and purified by IMAC. The Sal-1-Srt-His6 protein (from now on denoted Sal-1) was a kind gift from Dr. Linnea Enstedt and was produced as described elsewhere.^10^ The extracellular protein domain (position 43–300) of human CD38 (UniProt ID: P28907) fused in the C-terminal to the amino acid sequence ENLYFQG*EDQVDPRLIDGK* (underlined sequence, TEV protease recognition sequence; italicized sequence, HPC4 tag) was kindly provided by Dr. Hanna Tegel. This protein is from now on termed CD38 (for sequences, see Supporting Information Table S1).

### 2.3 Production of G_5_-Silk Nanowires

Nanowires (30 μm in length, ∼1 μm in diameter) of G_5_-silk were produced by laterally dragging a droplet of soluble G_5_-silk (0.4 mg/mL) over a superhydrophobic pillar surface (pillar distance = 30 μm), a procedure described in more detail elsewhere.^27^ Produced silk nanowires were detached from the pillar surface and released into 50 mL of 70% ethanol (v/v) using an ultrasonic bath for 3 min. To concentrate the nanowires, centrifugation (7000 rcf) for 30 min was performed, followed by removal of ∼45 mL of the supernatant. To exchange the 70% ethanol for a physiological buffer, three consecutive centrifugations (7000 rcf, 30 min) were performed, removing the ethanol supernatant and adding 50 mM Tris/0.001% Tween 20 after each centrifugation step. The final G_5_-silk nanowire batches (every batch was generated from four pillar surfaces) were each contained in 1–1.5 mL of 50 mM Tris/0.001% Tween 20 (Tris–T_0.001_).

### 2.4 Fiber Formation of G5-Silk

Macroscopic G_5_-silk fibers were produced by allowing the soluble G_5_-silk protein at 1 mg/mL to self-assemble at an air–liquid interface under cyclic expansion and compression for 3 h at room temperature. The procedure for fiber formation has been described in detail elsewhere.^28^ The fibers were washed in 20 mM Tris (pH 8) buffer and then stored in 20 mM Tris at +4 °C until use.

### 2.5 Sortase Coupling of G-Silk and G_5_-Silk in Solution

G-silk and G_5_-silk proteins in PBS buffer were dialyzed (Slide-A-Lyzer Dialysis Cassette, 2000 MWCO, Thermo Fisher Scientific) around 16 h at +4 °C against 1000 times excess of 20 mM Tris buffer (pH 8), to avoid coprecipitation of calcium and phosphate. Separate sortase coupling reactions in solution were performed for G-silk and G_5_-silk using Z-domain as a target protein. For each individual sortase coupling reaction, silk protein and Z-domain were added in equimolar concentrations of 17 μM in Sortase Ligation Buffer (50 mM Tris, 150 mM NaCl, 10 mM CaCl_2_, pH 7.6–7.8), together with 3 μM sortase. The sortase coupling reactions were allowed to proceed at room temperature for a total of 3 h. At specific time points (1–2, 30, 60, 120, 180 min) during the coupling reactions, a 40 μL sample was taken out from each reaction solution and immediately mixed with 10 μL of 5× protein gel loading dye (85% (v/v) glycerol, 20% (w/v) SDS, β-mercaptoethanol, 1% (w/v) bromophenol blue) and heated at 95 °C for 5 min to stop the sortase reaction. The samples were then separated using SDS-PAGE (NuPAGE 4–12% Bis–Tris, Invitrogen, Waltham, MA), which was subsequently stained with Coomassie Brilliant Blue. The same procedure as described above was followed for the sortase coupling of G-silk and G_5_-silk to the target protein scFv_CD38_.

### 2.6 SPR Using Silk Coatings

ProteOn sensor chips (GLM or GLC, Bio-Rad Laboratories, Hercules, CA) were prepared for experiments by performing four consecutive cleaning steps constituted by formic acid, TL1 wash, plasma treatment, and TL1 wash in order to remove the surface functionalizations (alginate and carboxyl groups) on the chip, leaving a bare gold surface. The washing steps were done after removal of the sensor prism from the plastic holder. First, the sensor chip was immersed in formic acid (98%) for 10 min at room temperature, followed by extensive washing in Milli-Q water. Second, a TL1 wash was performed by immersing it in a mixture of 18 mL Milli-Q water, 3 mL H_2_O_2_ (30%), 4 mL NH_3_ (25%) for 10 min at 80 °C, followed by extensive washing of the chip in lukewarm Milli-Q water. Third, the chip was blow-dried in nitrogen gas and then plasma treated for 5 min using a plasma surface treatment instrument (Pico, Diener electronic GmbH). Fourthly, a TL1 wash was again performed on the chip. The cleaned sensor chip was stored in 99.5% ethanol at room temperature until further use. A newly cleaned sensor chip with no alginate or functional groups was used for each individual experiment.

For the ProteOn experiments with online monitoring of both sortase coupling and IgG binding, separate sensor chips were used for G-silk and G_5_-silk, respectively, and the experiments were performed on different occasions. For each experiment, a cleaned sensor chip was removed from the 99.5% ethanol, extensively rinsed in Milli-Q water, and blow-dried in nitrogen gas. A silk coating (G-silk or G_5_-silk) was generated by applying 700 μL of silk solution (0.1 mg/mL) onto the sensor chip surface and incubated for 30 min at room temperature, followed by extensive washing in 20 mM Tris (pH 8) or PBS (pH 7.4) buffer. The sensor prism was then quickly blow-dried in nitrogen gas and then immediately reattached to the ProteOn sensor chip plastic holder, followed by docking the chip into the ProteOn instrument (ProteOn XPR36 Protein Interaction Array System, Bio-Rad Laboratories). To achieve a stable baseline, a liquid flow of sortase ligation buffer at 100 μL/min was allowed overnight over the sensor chip. A sortase coupling solution consisting of 4.3 μM Z-domain and 0.06 μM sortase A in Sortase Ligation Buffer was flown over the silk coating (G-silk or G_5_-silk) in the ProteOn instrument at 25 μL/min, and the response was monitored for 30 min. In the next step, IgG purified from rabbit serum (I5006, Sigma-Aldrich, Stockholm, SE) in PBS at 100 nM was flown over the sensor surface, and the response was monitored for 30 min. As controls, a few sensor channels were used for flowing buffer only, both for the sortase coupling step and for IgG binding. Data was plotted as representative graphs that have been displaced in response (i.e., in height) in relation to each other in order to display zero response at *t* = 0.

For the ProteOn experiment analyzing the CD38 analyte binding to scFv_CD38_-functionalized G_5_-silk, the sortase coupling was performed outside the ProteOn instrument, prior to monitoring of CD38 binding using the ProteOn. The sensor chip was treated, as described in the previous section, to allow assembly of a silk coating using 700 μL of G_5_-silk (0.1 mg/mL) for 30 min at room temperature, followed by extensive washing with 20 mM Tris (pH 8). Without allowing the silk coating to dry between the steps, sortase coupling was performed by adding 4.3 μM scFv_CD38_ and 0.06 μM sortase in Sortase Ligation Buffer/0.001% Tween 20 to the sensor chip and incubates for 45 min at room temperature, followed by extensive washing with Tris–T_0.01_. The sensor chip was quickly blow-dried using compressed air and then immediately reattached to the ProteOn sensor chip plastic holder, followed by docking the chip into the ProteOn instrument. A stable baseline was achieved by an overnight flow over a chip of PBS (pH 7.4)/0.01% Tween 20 (PBS–T_0.01_) at 100 μL/min. For the analyte binding, 200 mM CD38 (extracellular domain) in PBS–T_0.01_ was flown over the sensor surface at 25 μL/min and the corresponding response was monitored for 30 min. As controls, a few sensor channels were used for the flowing buffer only. Data was plotted as representative graphs that have been displaced in response (i.e., in height) in relation to each other to display zero response at *t* = 0.

### 2.7 Sortase Coupling of the Z-Domain to G_5_-Silk Nanowires

G_5_-silk nanowires in 50 mM Tris–T_0.001_, generated from a total of eight pillar surfaces, were concentrated by 10 min centrifugation (13,000 rcf, +4 °C), and the liquid supernatant was removed. Coupling was performed in 200 μL of sortase reaction mix containing 17 μM Z-domain and 0.3 μM sortase A in Sortase Ligation Buffer, supplemented with 0.001% Tween 20 for 1 h at room temperature. For controls without sortase enzyme and/or Z-domain, equal volumes of Milli-Q water were added. The nanowires were washed 1× in 800 μL and 5 × 10 min in 750 μL Tris–T_0.001_ as described above.

For visualization of the functionalized G_5_-silk nanowires, the samples were stained with 0.05 mg/mL rabbit antimouse IgG-Alexa Fluor 488 (Thermo Fisher Scientific) in PBS–T_0.001_ for 30 min at room temperature while keeping in the dark. After resuspension in 2× volumes of PBS–T_0.001_ followed by 10 min incubation (dark), the samples were washed 4× as described above. Nanowire samples were then prepared by air-drying 5 μL of each sample onto a microscope glass slide followed by visualization using an inverted fluorescence microscope (Nikon Eclipse Ti) with excitation at 455–490 nm and emission at 500–540 nm. Micrographs were captured at 40× with an Andor Zyla camera using NiS element BR software.

### 2.8 Sortase Coupling of Sal-1 to Fibers of G_5_-Silk

The sortase coupling reactions were performed in polystyrene 96-well plates (655161, Greiner BIO-ONE, Kremsmünster, Austria), preblocked with 0.01% Tween 20 for 1 h at room temperature to prevent unspecific binding of the target protein and the sortase enzyme to the walls of the wells during the sortase coupling. Each G_5_-silk fiber (produced as previously described in Section 2.4) was divided in half, where each fiber piece was used as a replicate in the experiment. Following blocking, the silk fibers were added to the wells and immersed in the sortase coupling solution (4.3 μM Sal-1 and 0.06 μM sortase in Sortase Ligation Buffer/0.001% Tween 20). For controls without Sal-1 or sortase, the respective constituent was replaced by Milli-Q water. The coupling reactions were performed for 45 min at room temperature, followed by extensive washing of the silk fibers with 50 mM Tris–T_0.01_. The biochemical assay used for detection of coupled Sal target proteins was performed by first transferring the fibers to empty wells in the 96-well plate and then immersing the fibers in Fluorescein Di-β-d-Galactopyranoside (FDG, Invitrogen), 5 μg/mL in PBS, for 2 h at room temperature (dark). A plate reader (ClarioStar; BMG Labtech, Ortenberg, Germany) was used to measure the enzymatic activity of coupled Sal-1 every 30 min using excitation at 490 nm and emission at 525 nm. The fluorescence data was plotted as mean fluorescence (*n* = 2) ± standard deviation.

### 2.9 Statistics

RM 2-way ANOVA followed by Tukey’s test for multiple comparisons was performed to determine any statistical differences between the test group and control groups at each time point. *P* < 0.05 was considered significant.

## 3 Results and Discussion

In this paper, we investigated the approach to functionalize silk by coupling target proteins to silk in different formats, i.e., solution, coatings, nanowires, and macroscopic fibers. We selected protein domains of different sizes and functions to covalently bind to the silk by sortase-mediated coupling. The achieved function/activity of the resulting silk was then evaluated by affinity or enzyme activity assays.

### 3.1 Sortase Coupling of Target Proteins to Silk in Solution Is More Efficient with G_5_-Silk

**Figure 1:**
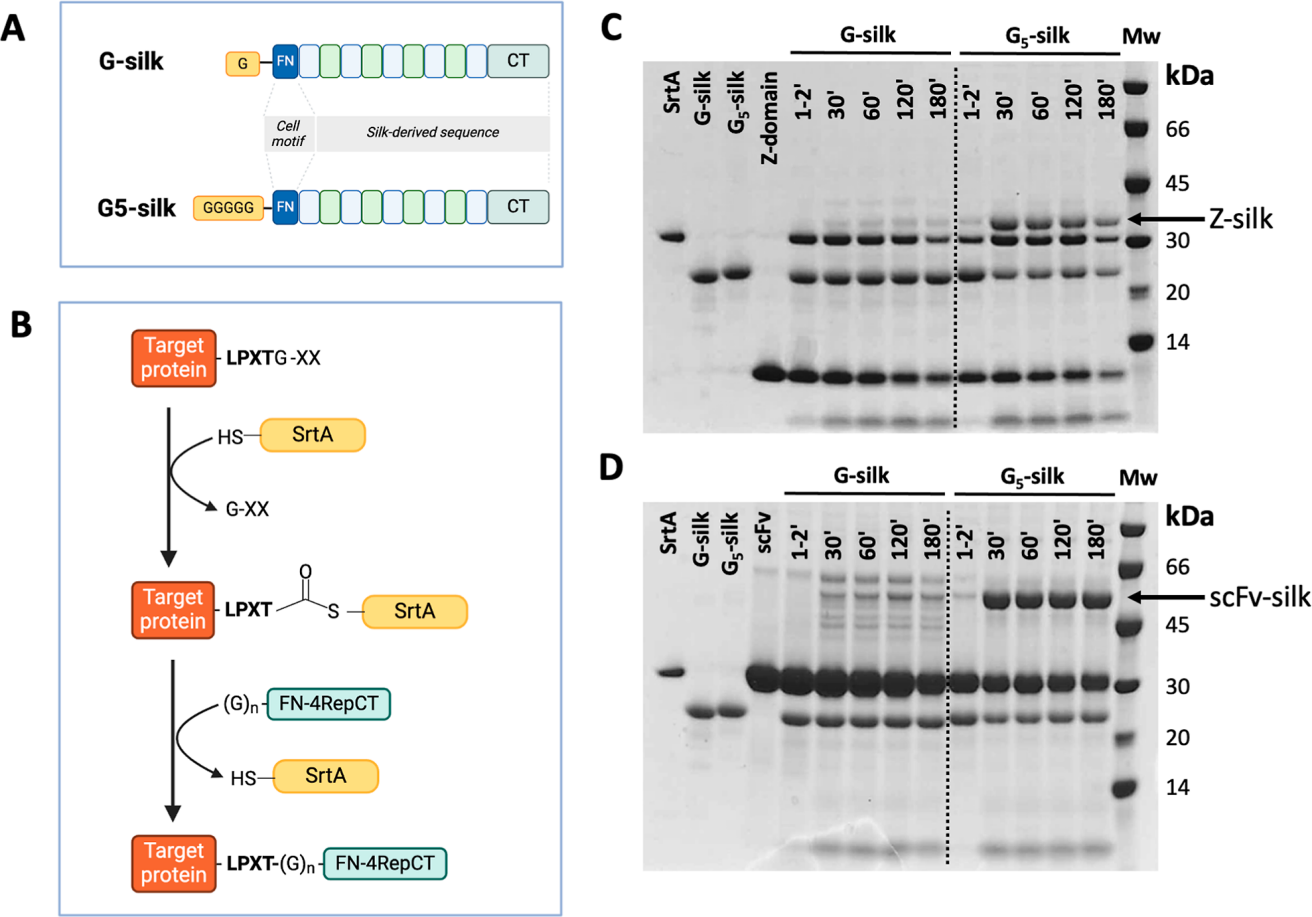
Sortase-mediated coupling of target proteins to silk proteins. (A) Schematics of the previously reported G-silk (FN-4RepCT), that naturally possesses one glycine in the N-terminus after production, and G_5_-silk, that has been designed in this study to contain five glycines at the N-terminus. (B) Schematics of the covalent coupling of a target protein to FN-4RepCT silk proteins (G-silk or G_5_-silk) using sortase A (Srt A). For the sortase coupling to occur, the target protein should possess a C-terminal sortase recognition motif (here, LPETGG). (C) SDS-PAGE analysis of sortase A-mediated coupling reactions of G-silk (left panel, 23 kDa) and G_5_-silk (right panel, 24 kDa), respectively, and the target protein Z-domain (Z-Srt-His, 9 kDa). During the reaction, samples were withdrawn, and the reaction immediately stopped by heating, after 1–2, 30, 60, 120, and 180 min. (D) SDS-PAGE analysis of corresponding sortase reactions with G-silk (left panel) and G_5_-silk (right panel) using another target protein, the antibody fragment scFv_CD38_ (scFv_CD38_-Srt-His6, 28 kDa). As a reference, individual proteins are shown to the far left in the gel image, and a low molecular weight protein marker M in kDa to the right.

To evaluate the efficiency of sortase coupling to silk in solution, the silk proteins G-silk (G-FN-4RepCT, 23 kDa) and the newly designed construct G_5_-silk (G_5_-FN-4RepCT, 24 kDa) with five N-terminal glycines (Figure 1A) were used for coupling to the target proteins (Figure 1B). The G_5_-silk was designed considering previous reports showing that two to five N-terminal glycines enhance sortase coupling.^29^ Both constructs also harbor a cell-binding motif from FN. Here, the selected target proteins were an IgG-binding Z-domain (Z-Srt-His6, 9 kDa) and a single-chain variable antibody fragment specific to CD38 (scFv_CD38_-Srt-His_6_, 28 kDa). SDS-PAGE analysis of the reaction mixtures, containing the target protein, the silk protein, and the sortase enzyme sampled at different time points (1–2, 30, 60, 120, and 180 min), confirmed the expected product formation. Both Z-silk band (∼32 kDa) and the scFv-silk band (∼51 kDa) were present for G-silk as well as for G_5_-silk reaction mixtures (Figure 1C,D), although clearly stronger bands were seen for G_5_-silk, indicating larger amount of product. Noteworthy, the coupling product of G_5_-silk was visible already at the first time point (i.e., 1–2 min after mixing of all reaction components), indicating an instant coupling reaction with both target proteins. In previous reports, Ton-That and co-workers observed a delay for several minutes in substrate cleavage by sortase immediately after mixing the reaction components.^30^ In the same study,^30^ an N-terminal addition of NH_2_-Gly, NH_2_-Gly_2_, or NH_2_-Gly_3_ to the original sequence increased the rate of substrate cleavage, wherein the fastest was observed with NH_2_-Gly_3_. The increased reaction rate for G_5_-silk compared to G-silk observed in the present study supports this previous finding. When comparing product amounts (as represented by the area and stain intensity of the protein band on the SDS-gel, Figure 1C,D), the product bands for G_5_-silk were much more pronounced compared to the corresponding product bands from G-silk. Image analysis (using the software ImageJ) of the band intensities of the product band normalized to that from G-silk at 1–2 min was compared. The signal for product bands from G_5_-silk couplings was higher than those from G-silk, for both Z-domain and scFv_CD38_ (Figure S1 in the Supporting Information). The results are in line with the study by Mao and team,^31^ which demonstrated the rate of sortase coupling was higher with two or more glycines at the N-terminus.

We also observed a peak of the coupling reactions after 30 min with both target proteins to G-silk/G_5_-silk (Figure 1C,D), similar to the preferred 30 min reactions in previous studies by other groups.^24,31,32^ Furthermore, it can be observed that the amount of the Z-silk product formed from G_5_-silk had decreased at 180 min compared to 30 min. This is most probably due to silk self-assembly or aggregation of the Z-silk protein over time at room temperature, concurrent with a more dim solution, resulting in a decrease in soluble protein concentration. This observation is clear from Figure S1a in the Supporting Information, showing that the product Z-silk reduced over time. In general, the coupling with sortase A was reproducible, also for a similar silk protein, 4RepCT, with one N-terminal Glycine and no FN-motif (Figure S2 in the Supporting Information). Taken together, these results show that the sortase coupling to silk in solution using two different target proteins is possible for both G-silk and G_5_-silk. The finding that G_5_-silk gives a more efficient coupling reaction verifies that having five N- terminal glycines for sortase coupling is both efficient and rapid, compared to having only one glycine, also for silk proteins.

### 3.2 Sortase Coupling to Silk Coatings Is More Efficient with G_5_-Silk

Here, we investigated whether the results from sortase coupling of target proteins to silk proteins in solution can be translated to silk coatings. Depending on the specific application in mind, the choice of material format of silk can be important. Silk coatings have previously been shown to form spontaneously by self-assembly onto surfaces of various materials like plastics, metals, and natural biopolymers.^33−36^ For that reason, the coating format is of interest for applications where it is desired to cover a surface with a functional moiety, for example, a functional coating on a medical implant, an antimicrobial coating, or a coating with affinity properties on a biosensor surface.

**Figure 2:**
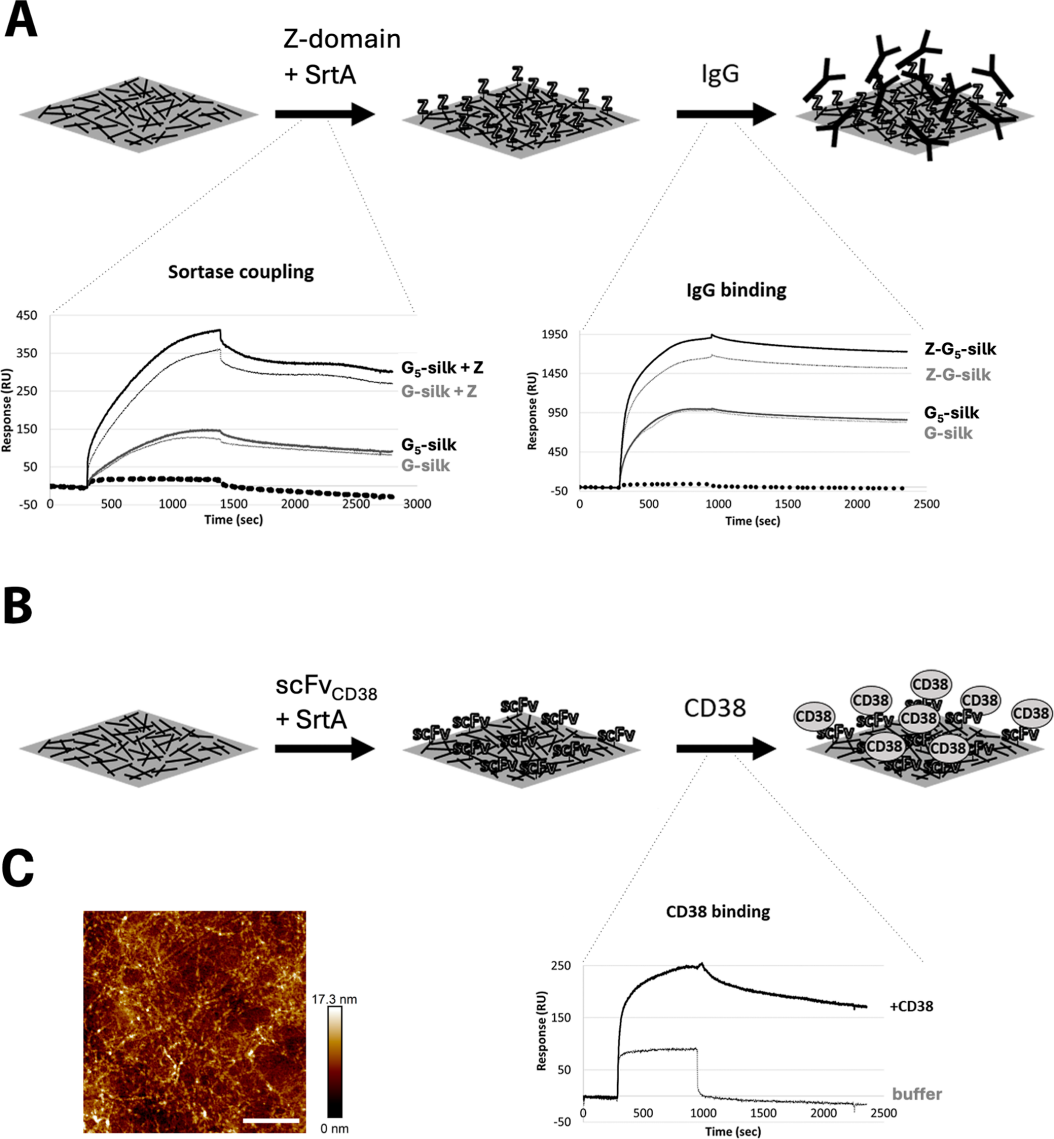
Sortase coupling of affinity domains to silk coatings, and evaluation of the resulting binding functionality. (A) Schematics of the principle for generating a functionalized silk coating consisting of a network of self-assembled G-silk and G_5_-silk fibrils on a surface using sortase A (Srt A). Sortase coupling of the target protein Z is achieved by immersing the silk coating in a sortase reaction mixture containing both sortase enzyme and target protein. To test the binding functionality, the corresponding analyte (i.e., IgG) is added to the functionalized silk coating, and the actual binding can be monitored using SPR technology. In the left SPR sensorgram, sortase coupling of the Z-domain is evaluated by SPR comparing G-silk and G_5_-silk (upper curves). As controls, the corresponding SPR curves representing sortase coupling without Z-domain are shown (middle curves), as well as the response from only buffer (lower, dotted curve). In the right sensorgram, the corresponding binding of IgG is presented for G-silk and G_5_-silk, with (upper curves) and without (middle curves) coupled Z-domain. The lower (dotted) control curve shows the response for only buffer (no IgG). (B) Schematics of the strategy for generating a sortase-mediated functionalized G_5_-silk coating using scFv_CD38_ as the target protein. Here, sortase-mediated coupling of scFv_CD38_ is done on the chip before being connected to the SPR instrument. The sensorgram shows the binding response from CD38 to scFv_CD38_-silk (upper curve). As a reference, the corresponding response from the flowing buffer only is presented (lower curve). (C) AFM scan of a G-silk coating on polystyrene, showing its nanofibrillar structure. Scale bar: 1 μm.

The strategy to investigate the functionalization of silk coatings with a target protein using sortase coupling is outlined in Figure 2A (upper panel). For this, we used a recently published method to create stable nanofibrillar silk surface coatings from soluble silk proteins^34^ and applied it using G-silk and G_5_-silk on gold surfaces. This coating procedure has previously been shown to yield a mesh of fibrils of nanodimensions (60–400 nm long, 8–20 nm wide). In the present study, the solid surface chosen was a SPR sensor chip that had been cleaned to remove any functionalization, leaving the bare gold surface, as described in Experimental Section 2.6. Sortase coupling of the IgG-binding Z-domain to the G-silk and G_5_-silk coatings was performed and monitored in real time by SPR measurements (Figure 2A, left sensorgram). A significant signal was shown, compared to the controls without a Z-domain present, suggesting that the Z-domain was efficiently coupled to G-silk and G_5_-silk coatings. Further, the following binding of IgG to the Z-G-silk and Z-G_5_-silk coatings was analyzed by SPR, and the corresponding sensorgram showed a clear response for both types of Z-silk coatings (Figure 2A, right sensorgram). The results confirmed that also for a premade silk material, as a coating, it is possible to covalently couple a target protein to the silk material using sortase and retain the functionality of the target protein.

When comparing the responses for sortase coupling of the Z-domain to G-silk and G_5_-silk (Figure 2A, left sensorgram), we observed that the response is slightly higher for the G_5_-silk, suggesting that more Z molecules had been coupled to the G_5_-silk coating. This, in turn, indicates that also for the coating format, the G_5_-silk variant is most efficient for sortase coupling, in the same manner as observed when performing sortase coupling in solution (see results in Section 3.1). The difference in coupling efficiencies between G-silk and G_5_-silk could be due to better accessibility and higher flexibility provided by the oligoglycine linker in G_5_-silk. A comparative study on sortase coupling of eGFP to solid surfaces by Chan and colleagues^24^ reported that the tetra-glycines on glycidyl methacrylate beads showed a rapid and high degree of coupling to eGFP compared to mono- or diglycine beads. This suggests that oligoglycines at the N-terminus improve sortase coupling efficiency not only in solution but also on solid surfaces like coatings.

The control coatings without a Z-domain during the sortase coupling reaction should in theory lack IgG-binding capacity. Yet, they show some binding of IgG, detectable both with G-silk and G_5_-silk (Figure 2A, right sensorgram). The responses could be attributed to unspecific adherence of the sortase enzyme to the silk coatings (Figure 2A, left sensorgram) and further unspecific binding of IgG to the adhered sortase enzyme or to the silk coatings themselves (Figure 2A, right sensorgram). Some of the nonspecific binding of sortase to silk was washed off during the dissociation step, and the true coupling to Z-silk remained, as seen from Figure 2A, left sensorgram. For comparison, the response curve from flowing only buffer over a silk coating is also presented to show the drift in the SPR system (Figure 2A, left sensorgram, lower/dotted response curve). The β-sheet-rich structure of assembled silk is likely prone to mediate unspecific protein adhesion. It has previously been shown that silk proteins tend to bind nonspecifically to polyclonal antibodies and to horse radish peroxidase-conjugated IgGs, but this unspecific interaction was shown to be minimal compared to the specific reactivity detected for functionalized silk with respective ligands.^37^ Sato et al. developed a novel immune detection system using scFv-conjugated silk fibroin proteins with sensitivity matching the conventional systems even with the above-mentioned nonspecific binding of silk to IgGs.^37^

To show the generality of the sortase coupling approach also with larger molecules to silk coatings, the experiment was repeated with the antibody fragment scFv_CD38_ (28 kDa) (Figure 2B, upper panel). As the hitherto presented data from sortase coupling of target proteins to silk proteins, both in solution (Z-domain and scFv), and as silk coatings (Z-domain), indicated that G_5_-silk is most efficient, G-silk was omitted in further experiments. For these experiments, the G_5_-silk-coated SPR sensor chip was subjected to sortase coupling of scFv_CD38_ before being connected to the SPR instrument. The sensor chip was then docked into the instrument and binding of the analyte CD38 was measured by SPR. A binding response higher than the buffer control demonstrated that scFv_CD38_ has preserved affinity to CD38 also after coupling to silk coatings. Thus, the results indirectly confirmed a successful sortase coupling of scFv_CD38_ to coatings of G_5_-silk (Figure 2B). To the best of our knowledge, this is the first study reporting a covalent conjugation of scFv fragments to silk coatings. To visualize the nanofibrillar silk coating, an AFM micrograph is shown in Figure 2C. These data establish the potential of the sortase coupling as a general strategy for covalent functionalization of G_5_-silk coatings with small (Z-domain) and larger (scFv fragment) proteins.

### 3.3 Sortase Coupling Can Be Used for Attachment of a Target Protein to Nanowires of G_5_-Silk

**Figure 3:**
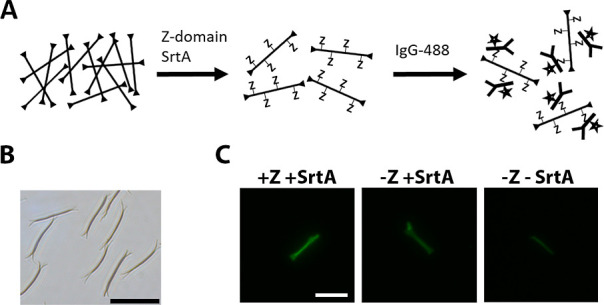
Use of sortase to covalently attach the Z-domain to nanowires of G_5_-silk. (A) Schematics showing the strategy for functionalizing G_5_-silk nanowires with the target protein Z. Sortase coupling was performed by mixing concentrated nanowires with the Z-domain and sortase enzyme. After washing, functionalized nanowires were visualized by the addition of an IgG-fluorophore (IgG-488) and subsequent fluorescence microscopy analysis. (B) Bright-field microscopy image showing a representative appearance of silk nanowires in suspension, after release from the pillar surface. The nanowires were produced from a surface with a pillar interspacing of 30 μm. Scale bar represents 30 μm. (C) Fluorescence microscopy images of G_5_-silk nanowires after the sortase coupling of the Z-domain and subsequent addition of IgG-Alexa Fluor 488 (IgG-488) followed by washing. Coupling controls without the addition of Z-domain and without Z-domain and sortase are shown. Scale bar represents 25 μm.

For some applications, it might be more suitable to use other material formats of silk than the two-dimensional coating. Therefore, the format of three-dimensional silk nanowires was used to investigate if it is possible to covalently attach a target protein to freestanding silk formats by sortase coupling. The production of nanowires based on the 4RepCT protein has been recently reported^12,27^ and is described in Experimental Section 2.3. Briefly, nanowires of G_5_-silk were produced by dragging a droplet of the soluble G_5_-silk along a surface with superhydrophobic pillars. The shear forces arising from dragging the drop induce self-assembly of the silk solution, forming ultrathin silk wires between the pillars. The nanowires were then released from the pillar surface in 70% ethanol, followed by buffer exchange and concentration by using several rounds of centrifugation. Thereby, nanowires with dimensions of 30 μm in length and ∼1 μm in diameter were obtained. The strategy for functionalization of the released G_5_-silk nanowires using sortase is presented in Figure 3A. In the first step, a solution with released nanowires (Figure 3B) was mixed with the target protein Z and the sortase enzyme for the coupling reaction to occur, followed by a wash. In the second step, a fluorophore-labeled IgG (IgG-Alexa Fluor 488) was added and allowed to bind to the coupled Z molecules. To visualize the nanowires, bright-field and fluorescence microscopy was used.

**Figure 4:**
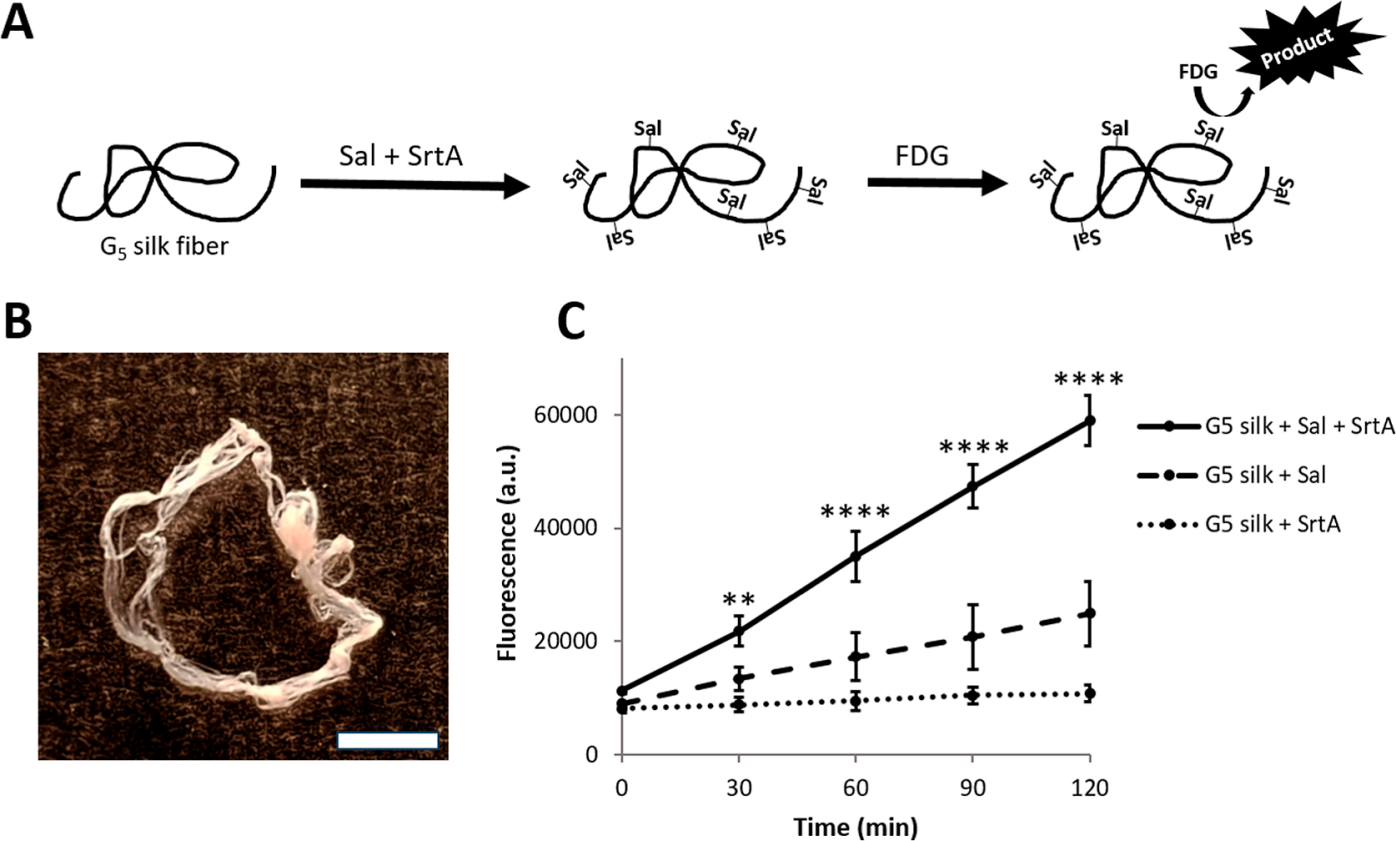
Construction and functional evaluation of Sal-1-silk fibers by sortase coupling. (A) Schematics showing functionalization of G_5_-silk fibers performed by sortase coupling to covalently attach the target endolysin Sal-1 (a peptidoglycan-degrading enzyme) to the fibers. Functional readout was done using a biochemical assay in which an added FDG substrate is converted into a fluorescent substrate by Sal-1 coupled to fibers. (B) Bright-field microscopy image of a G_5_-silk fiber, subsequently used for sortase coupling of Sal-1. Scale bar represents 2 mm. (C) Graph plot presenting values from the fluorescence measurements during the biochemical assay, in which the added FDG substrate is converted into a fluorescent product by the Sal-1 enzyme. For each condition, the fluorescence values at each measured time point represent the total activity of all Sal-1 molecules present on the respective fiber. Significant differences between Sal-silk (G_5_-Silk + Sal + Srt A, solid line) and the Sal-1-lacking control (G_5_-Silk + Srt A, dotted line) are indicated, ** for *P* < 0.01 and **** for *P* < 0.0001. Not indicated in the plot are the significant differences detected between Sal-Silk (G5 silk + Sal + Srt A, solid line) and the Sortase-free control (G5 silk + Sal, dashed line) at 60 min (*p* < 0.001), 90, and 120 min (*p* < 0.0001).

For G_5_-silk nanowires with both Z-domain and the sortase enzyme present during the coupling reaction, there is a clear green fluorescence signal from IgG-488 (Figure 3C). This indicates that the Z molecules have been covalently coupled to the silk and, in the following step, have bound to IgG-488. In contrast, the control lacking the sortase enzyme during the coupling reaction showed a very faint fluorescence signal, suggesting that the Z-domains did not adhere spontaneously onto the nanowires and, in turn, could not capture IgG-488. On the contrary, the control that lacked the Z-domain, but had sortase present during the coupling reaction, showed a fairly pronounced fluorescent signal compared to the other two controls. It was, however, clear that this signal was less intense compared to nanowires with a sortase-coupled Z-domain, which showed a stronger response (Figure 4C, SF3). The reason behind the fluorescence detected from the nanowire lacking Z-domain is likely due to unspecific protein adhesion to the β-sheet-rich silk structures in the silk nanowires. The potential for charge–charge interactions between sortase A and the nanowires is less likely, since they both carry net positive charges at the used conditions. Thus, the detected signal suggests some unspecific adherence of the sortase enzyme to the silk nanowires, and that the IgG-488 molecules then in turn bind nonspecifically to the adhered sortase enzyme, similar to the observations made with silk coatings in Section 3.2. Despite this unspecific binding, the difference in signal intensity detected between nanowires with and without the sortase-coupled Z-domain is statistically significant (Figure S3 in the Supporting Information). This suggests that the three-dimensional silk nanowires could be functionalized with a Z-domain to achieve IgG-binding properties using the sortase A enzyme.

### 3.4 Fibers of G_5_-Silk Can Be Functionalized with an Enzyme Using Sortase Coupling

Another interesting format for various biomedical applications is the silk fiber.^28^ Macroscopic (cm-long) silk fibers can be formed by allowing the G_5_-silk protein in solution to self-assemble into fibers at an air–liquid interface during a cyclic expansion/compression process.^28^ The cell-binding FN-4RepCT silk protein has previously been shown able to self-assemble into such macroscopic fibers also in the presence of mammalian cells, thereby integrating the cells into an extracellular matrix-like structure, constituted by the FN-silk fiber bundle, resulting in high cell viability and proliferation.^38^

In our previous study, the peptidoglycan-degrading endolysin Sal-1 was used for successful sortase coupling to coatings of 4RepCT silk, where coupled Sal-1 showed enzymatic activity after coupling.^10^ Herein, G_5_-silk in a macroscopic fiber format was instead used for sortase coupling of the Sal-1 enzyme (Figure 4A). After production of the G_5_-silk fibers (Figure 4B) and sortase coupling, the enzymatic activity of the attached Sal-1 enzymes was determined by adding the substrate FDG that is converted into a fluorescent product molecule by the Sal-1 enzyme and the signal intensity was monitored over time.

The fluorescence data show that G_5_-silk fibers with covalently coupled Sal-1 enzymes (Sal-1-silk) gave the highest rate of substrate conversion (Figure 4C), compared to the two control conditions lacking either sortase or Sal-1 during the sortase reaction. Some unspecific binding of Sal-1 to the fibers was observed, evident from the slight increase of endolysin activity (dashed line) over time. However, the activity level of Sal-1 covalently coupled to the silk (solid line) was significantly higher (*p* < 0.001) from 1 h reaction time and onward. The results indicate that a covalent coupling of Sal-1 to the G_5_-silk fibers was efficient and that the coupled Sal-1 enzymes maintained their activity after coupling. Similar results were reported by Hata and colleagues,^39^ where immobilizing the enzyme *Streptococcus bovis* 148 α-amylase on nanoparticle surfaces using sortase A maintained functionality. Moosavi and team have also reported maintained activity of *Candida antarctica* lipase B (CalB) when immobilized via sortase coupling to graphene oxide nanoparticles.^40^ The current study also demonstrates that silk can be used for sortase-mediated functionalization with an enzyme, thereby providing a proof of concept for applications of G_5_-silk fibers in the development of antimicrobial biomaterials.

## 4 Conclusions

In summary, we functionalized recombinant spider silk in the formats of coatings, nanowires, and macroscopic fibers with either of the three target proteins: the Z-domain, a single-chain variable fragment scFv_CD38_, and the endolysin Sal-1, using a sortase A coupling strategy. The presented data demonstrates the profit of additional glycines on the silk construct, when it comes to coupling efficiency during sortase reactions. The results showed that FN-4RepCT silk with five N-terminal glycines (G_5_-silk) gave the most efficient coupling. The covalently attached binders, Z-domain and scFv_CD38_, both retained their functional affinity after sortase coupling to silk. Furthermore, when the endolysin Sal-1 was coupled to the G_5_-silk fibers, its specific enzymatic activity was preserved. Through this work, we achieved a general approach for site-specific, covalent attachment of target proteins to G_5_-silk coatings, nanowires, and fibers under mild conditions using sortase A. This functionalization of various silk formats in a site-specific manner using sortase A could be useful for several biomedical applications such as targeted drug delivery, diagnostics, cell culture, and antibacterial biomaterials.

## Supporting Information Available

Data and analyses from additional coupling experiments (in solution and on nanowires) and amino acid sequences of the proteins used in the study (PDF)

## Abbreviations

FDG: fluorescein Di-β-d-galactopyranoside
Fc region: fragment crystallizable region
FN: fibronectin
G-silk: FN-4RepCT (with one N-terminal glycine)
G_5_-silk: G_5_-FN-4RepCT
His6: hexahistidine tag
IgG: immunoglobulin-G
IMAC: immobilized metal ion affinity chromatography
MaSp 1: major ampullate spidroin 1
scFv: single-chain variable antibody fragment
SDS-PAGE: sodium dodecyl sulfate polyacrylamide gel electrophoresis
SPA: staphylococcal Protein A
SPR: surface plasmon resonance
Srt: sortase recognition tag
Srt A: sortase A enzyme
TEV protease: tobacco etch virus protease
Trx: thioredoxin
488: Alexa Fluor 488
