# Supplementary material for: Site-Specific Functionalization of Recombinant Spider Silk Using Enzymatic Sortase Coupling
Source: ACS Omega. 2025 Feb 6;10(6):5943–52. doi: 10.1021/acsomega.4c09900 (PMC11840766; doi:10.1021/acsomega.4c09900)
Supplement: Supplementary file 1 — ao4c09900_si_001.pdf [file ao4c09900_si_001.pdf]

SUPPORTING INFORMATION

Site-specific functionalization of recombinant spider silk using enzymatic sortase coupling

*Rajeev Pasupuleti, Ronnie Jansson, Ida Isacsson, Felicia Hogan, Mona Widhe, My Hedhammar**

KTH Royal Institute of Technology, School of Engineering Sciences in Chemistry, Biotechnology and Health, Department of Protein Science, AlbaNova University Center, Roslagstullsbacken 21, SE-106 91 Stockholm

*Corresponding author: myh@kth.se

Supplementary Figures

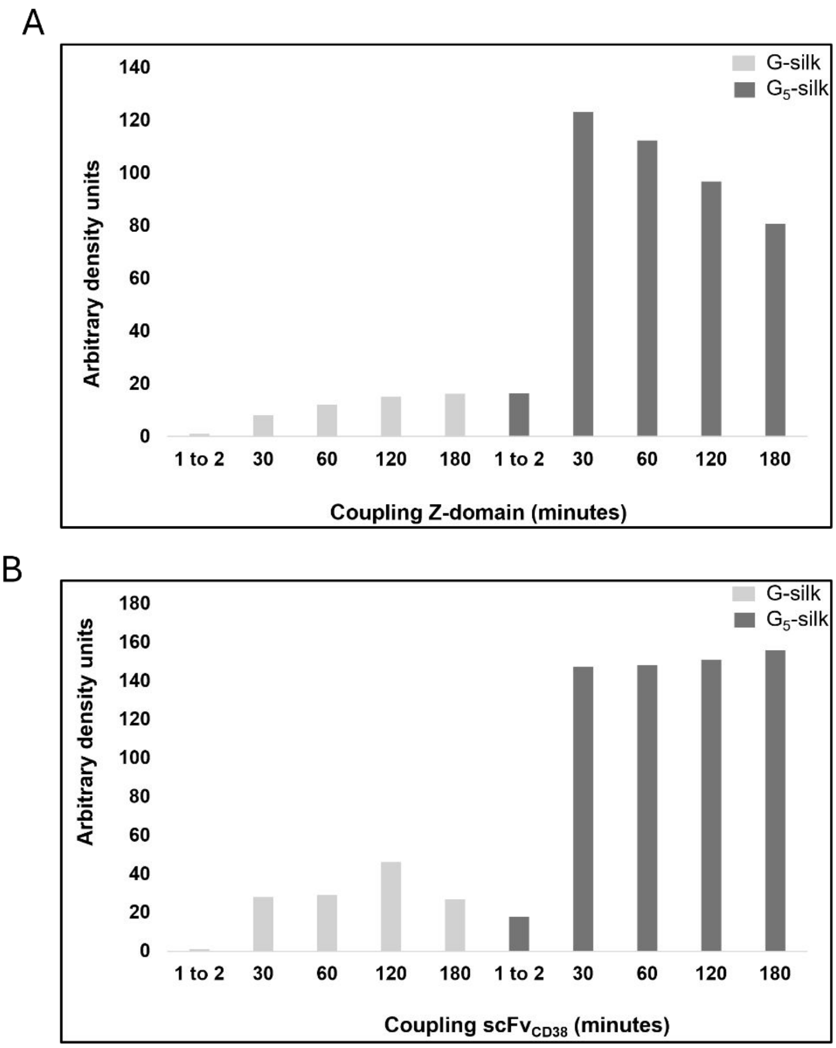

Figure S1: Comparison of Z-domain and $scFv_{CD38}$ coupling to G-silk and G5-silk using sortase. (A) coupling of Z-domain to G5-silk showed efficient coupling compared to G-silk at all tested time points. (B) Similar pattern was observed with $scFv_{CD38}$ coupling to G5-silk and was higher when compared to G-silk. The analysis was performed by peak area method using image J software[1]. The values were normalized to G-silk at 1 to 2 minutes.

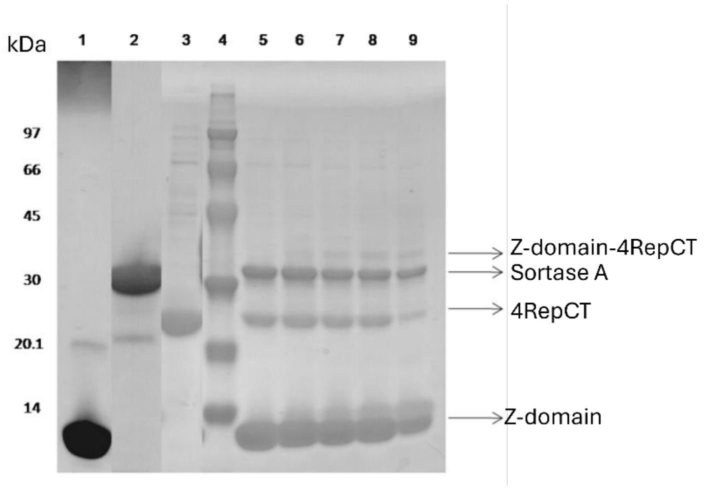

Figure S2 Sortase A mediated coupling in solution. Lane 1, Z-domain wildtype(8.79 kDa); lane 2, Sortase A wildtype (24.31 kDa); lane 3, 4RepCT (23 kDa); lane 4, Marker; lane 5, coupling in solution after 0 minutes; lane 6, after 1.5 hrs; lane 7, after 3 hrs; lane 8, after 6 hrs; lane 9, after 24 hrs.

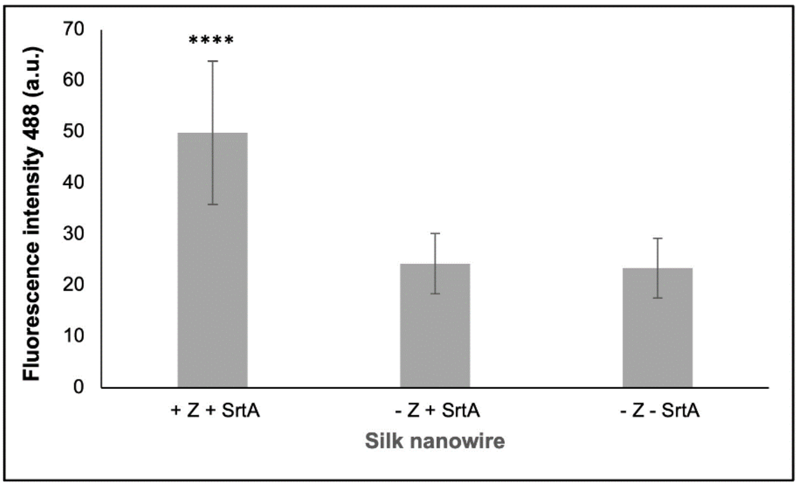

Figure S3 Analysis of Z-domain coupling to G_5-silk nanowires using sortase A, visualized by IgG-488. Image J[1] analysis showed higher IgG-488 signal for silk nanowires where silk, sortase A and Z-domain were all present during the sortase coupling reaction, compared to the controls lacking either Z-domain, or both Z-domain and sortase.. The corrected fluorescence was calculated by the formula= Integrated density – (Area of selected region x Mean fluorescence of background). The analysis was performed with data from three experiments with >6 replicates each.

Supplementary Tables

Table S1 Amino acid sequences of all proteins used in the study. N-terminal Glycine/s, bold; FN4RepCT, underlined; Sortase A, grey and underlined; Z-domain, italicized and underlined; hexahistidine tag, HHHHHH; Sortase -tag, LPETGG in grey; Sal-1, ; scFv$_{CD38}$, bold and underlined.

Protein details	Protein short name	Amino acid sequence
G-FN4RepCT	G-silk	**G**PNSCTGRGDSPACGSASGQGGYGGLGQGGYGQGAGSSAAAAAAAAAAAAGGQGGQGQGGYGQGSGGSAAAAAAAAAAAAAAAGRGQGGYGQGSGGNAAAAAAAAAAAAAAAGQGGQGGYGRQSQGAGSAAAAAAAAAAAAAAGSGQGGYGGQGQGGYGQSSASASAAASAASTVANSVSRLSSPSAVSRVSSAVSSLVSNGQVNMAALPNIISNISSSVSASAPGASGCEVIVQALLEVITALVQIVSSSSVGYINPSAVNQITNVVANAMAQVMG
GGGGG-FN4RepCT	G$_5$-silk	**GGGGG**ANSCTGRGDSPACGSASGQGGYGGLGQGGYGQGAGSSAAAAAAAAAAAAGGQGGQGQGGYGQGSGGSAAAAAAAAAAAAAAAGRGQGGYGQGSGGNAAAAAAAAAAAAAAAGQGGQGGYGRQSQGAGSAAAAAAAAAAAAAAGSGQGGYGGQGQGGYGQSSASASAAASAASTVANSVSRLSSPSAVSRVSSAVSSLVSNGQVNMAALPNIISNISSSVSASAPGASGCEVIVQALLEVITALVQIVSSSSVGYINPSAVNQITNVVANAMAQVMG
Sortase A	Srt A	MEYKLILNGKTLKGETTTEAVDAATAEKVFKQYANDNGVDGEWTYDDATKTFTVTEHMGSQAKPQIPKDKSKVAGYIEIPDADIKEPVYPGPATSEQLNRGVSFAEENESLDDQNISIAGHTFIDRPNYQFTNLKAAKKGSMVYFKVGNETRKYKMTSIRNVKPTDVEVLDEQKGKDKQLTLITCDDYNEKTGVWETRKIFVATEVKLEHHHHHH
Z-Srt-His6	Z domain	M*VDNKFNKEQQNAFYEILHLPNLNEEQRNAFIQSLKDDPSQSANLLAEAKKLNDAQAPK*VDGSGSGSLPETGGHHHHHH
scFv$_{CD38}$-Srt-His6	scFv$_{CD38}$	**GTQVQLVQSGAEVKKPGSSVKVSCKPSGGTFRSYAISWVRQAPGQGLEWMGRIIVFLGKVNYAQRFQGRVTLTADKSTTTAYMELSSLRSEDTAVYYCTGEPGARDPDAFDIWGQGTMVTVSSGGGGSGGGGSGGGGSDIQMTQSPSSLSASVGDRVTITCRASQGIRSWLAWYQQKPEKAPKSLIYAAS**

		**SLQSGVPSRFSGSGSGTDFTLTISSLQPEDFATYYCQQ**<br>**YNNYPLTFGGGTKVEIKRGR**GSGSGSLPETGGLEHHHH<br>HH
Sal-1-Srt-His6	Sal-1	MAKTQAEINKRLDAYAKGTVDSPYRIKKAT<br>SYDPSFGVMEAGAIDADGYYHAQCQDLITD<br>YVLWLTDNKVRTWGNAKDQIKQSYGTGFKI<br>HENKPSTVPKKGWIAVFTSGSYQQWGHIGI<br>VYDGGNTSTFTILEQNWNGYANKKPTKRVD<br>NYYGLTHFIEIPVKAGTTVKKETAKKSASK<br>TPAPKKKATLKVSKNHINYTMDKRGKKPEG<br>MVIHNDAGRSSGQQYENSLANAGYARYANG<br>IAHYYGSEGYVWEAIDAKNQIAWHTGDGTG<br>ANSGNFRFAGIEVCQSMSASDAQFLKNEQA<br>VFQFTAEKFKEWGLTPNRKTVRLHMEFVPT<br>ACPHRSMVLHTGFNPVTQGRPSQAIMNKLK<br>DYFIKQIKNYMDKGTSSSTVVKDGKTSSAS<br>TPATRPVTGSWKKNQYGTWYKPENATFVNG<br>NQPIVTRIGSPFLNAPVGGNLPAGATIVYD<br>EVCIQAGHIWIGYNAYNGNRVYCPVRTCQG<br>VPPNHIPGVAWGVFKGSSGPAALGSSGLPE<br>TGHHHHHH

Supplementary References

1. Schneider, C., Rasband, W. & Eliceiri, K. NIH Image to ImageJ: 25 years of image analysis. *Nat Methods* **9**, 671–675 (2012). https://doi.org/10.1038/nmeth.2089
